# Supplementary material for: Asynchronicity of deglacial permafrost thawing controlled by millennial-scale climate variability
Source: Nat Commun. 2025 Jan 2;16:290. doi: 10.1038/s41467-024-55184-z (PMC11697416; doi:10.1038/s41467-024-55184-z)
Supplement: Supplementary file 1 — Supplementary Information [file 41467_2024_55184_MOESM1_ESM.docx]

**Supplementary file**

**Asynchronicity of deglacial permafrost thawing controlled by millennial-scale climate variability**

Xinwei Yan, Xu Zhang*, Bo Liu, Huw T. Mithan, John Hellstrom, Sophie Nuber, Russell Drysdale, Junjie Wu, Fangyuan Lin, Ning Zhao, Yuao Zhang, Wengang Kang, Jianbao Liu*

*Corresponding authors: [xuang@bas.ac.uk](mailto:xuang@bas.ac.ac) (X. Zhang); liujb@pku.edu.cn (J.B. Liu)

**This file includes:**

Supplementary Table 1

Supplementary Figures 1 to 13

Supplementary References

**Supplementary Table 1: Ground surface temperature information for the Tibetan Plateau from model runs presented in this study.**

| Ground Surface Temperature | Annual  [°C] | Min  [°C] | Max  [°C] |
| --- | --- | --- | --- |
| piCTRL | -0.69 | -13.03 | 7.90 |
| CTRL | -5.63 | -18.08 | 4.80 |
| Hosing | -4.85 | -17.24 | 6.31 |

**Supplementary Figures 1-13**


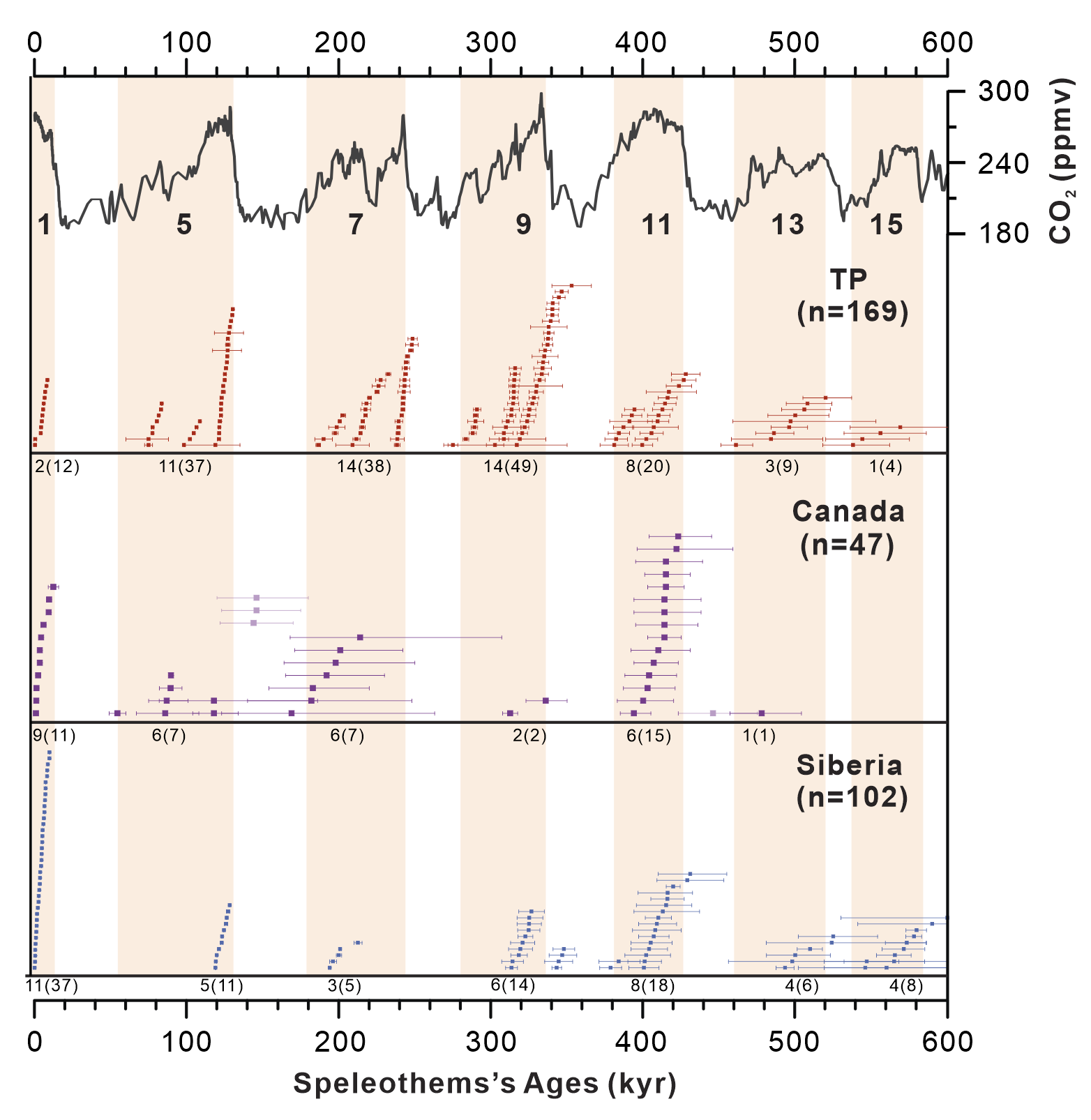


**Supplementary Fig. 1 | Compiled speleothem ages.** Individual U-Th/U-Pb ages with 95% uncertainties from published studies from Canadian, Siberian and Tibetan Plateau permafrost regions, the number below indicate the number of speleothems that grew and, in parentheses, the number of age determinations in each interglacial period(orange vertical bars). Also shown is the CO_2_ record of EPICA Dome C^1^, with interglacial Marine isotope stages (MIS) numbers below.


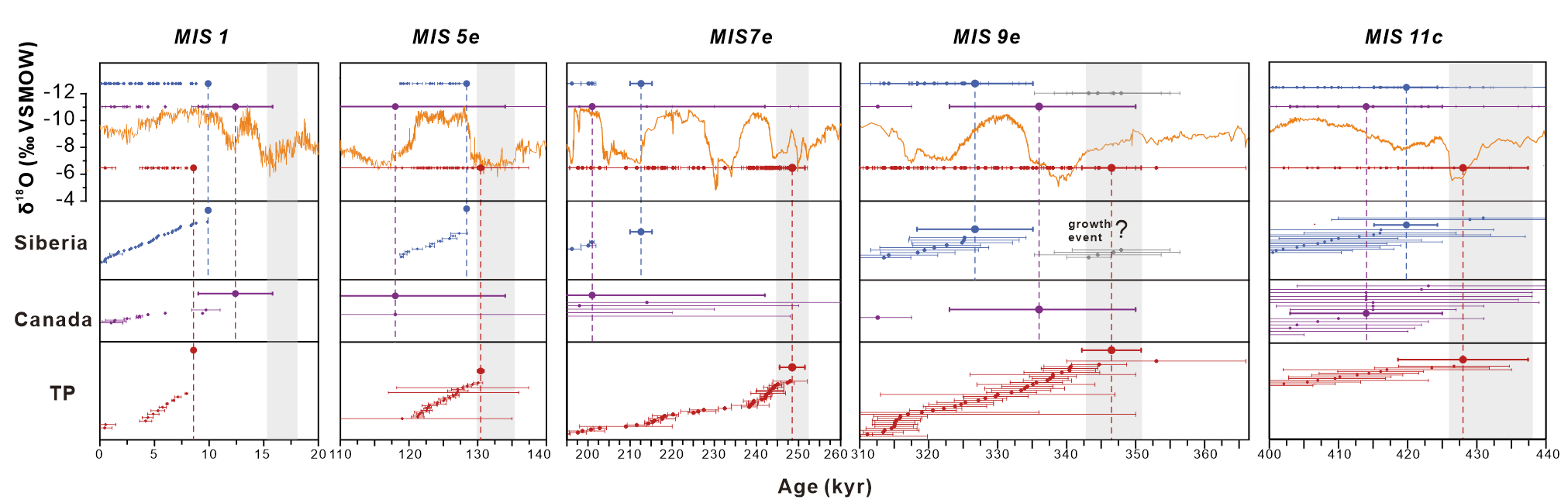


**Supplementary Fig. 2 |** **Selection of the dates representing onset of continuous thawing during interglacial MIS 1 to MIS 11c.** The panels show the distribution of Siberian (blue), Canadian (purple) and Tibetan Plateau (red) speleothem ages (±2σ) in time. The relatively large bold circle with error bars indicates the onset of continuous permafrost thawing in circumarctic (i.e. Canada and Siberia) and TP (Methods). The upper panel background is the East Asian Summer Monsoon composite records^2^ (orange). The grey error bars of T-IV represent a temporary growth event. Vertical bars indicate the Terminal stadials.


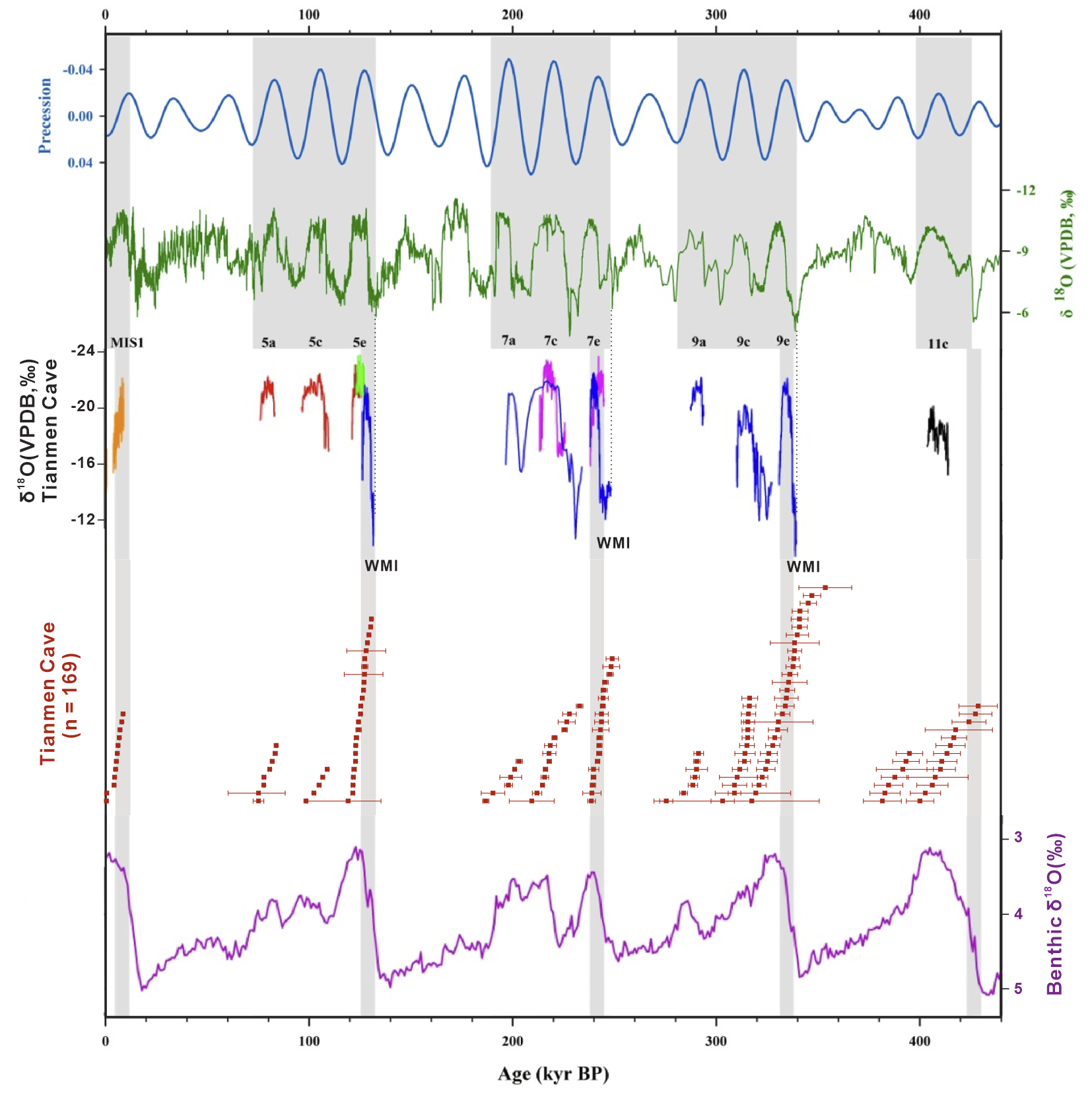


**Supplementary Fig. 3 |** **Comparison of Tianmen cave records with East Asian Summer Monsoon composite records.** From top to down, insolation precession^3^; East Asian Summer Monsoon composite δ^18^O records^2^; Tianmen cave δ^18^O record^4^, composed by six stalagmites in different colors, WMI indicates weak monsoon interval; ages with errors of Tianmen cave stalagmites; LR04 benthic δ^18^O stack^5^.


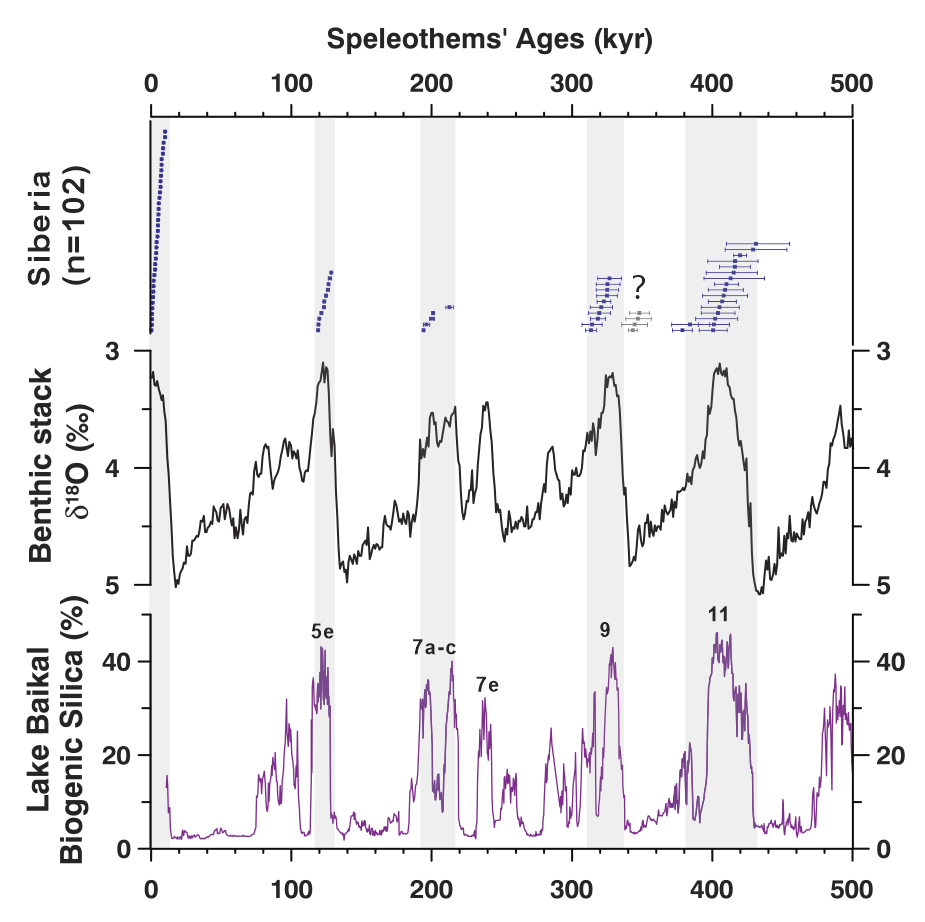


**Supplementary Fig. 4 |** **Comparison of speleothems’ growth periods in Siberia with benthic stack δ^18^O record**^5^ **and Lake Baikal biogenic silica contents**^27^**.** The grey error bars of T-IV represent a temporary growth event.


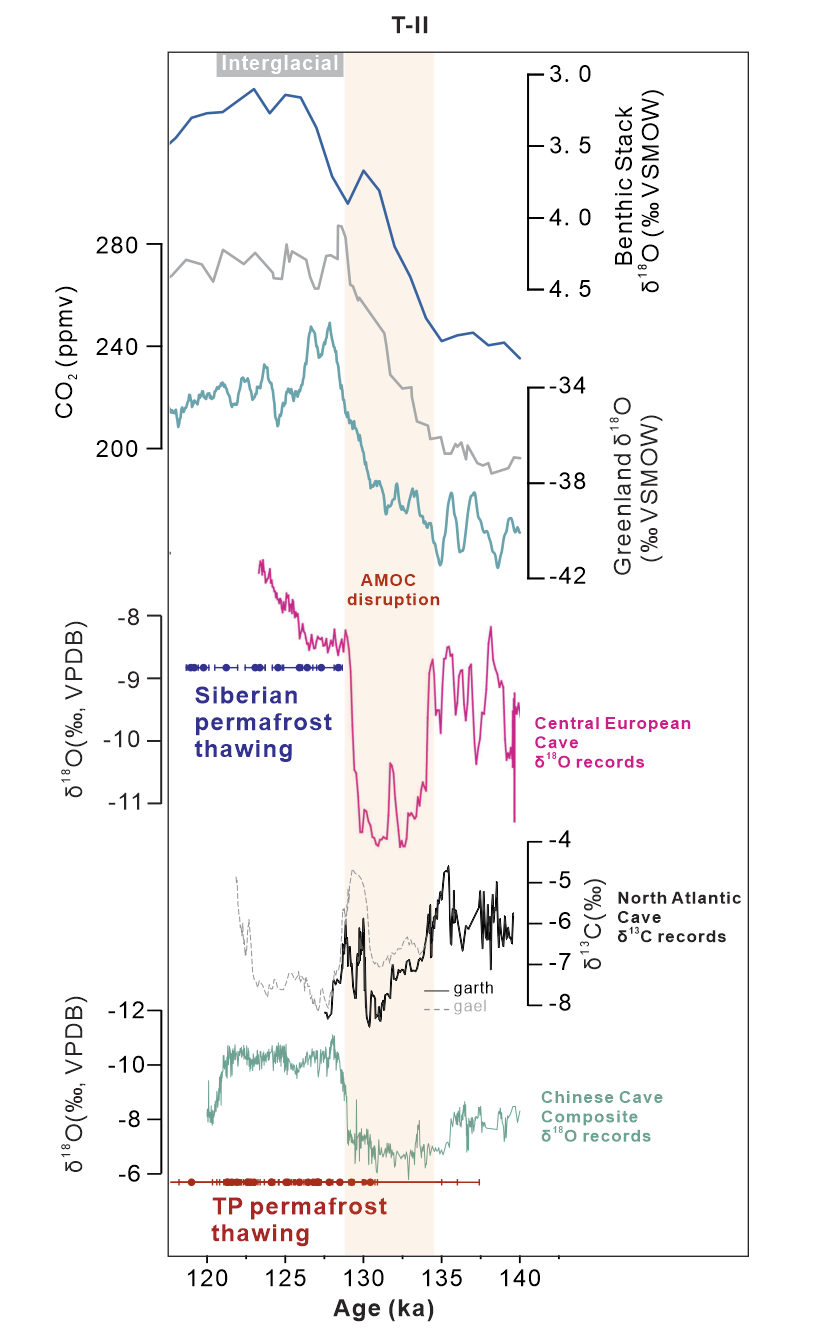


**Supplementary Fig. 5 |** **Comparison of speleothem growth phase with** **speleothem records during T-II, also shown is the global climatic background.** From top to bottom: Global benthic δ^18^O stack^5^; Atmospheric CO_2_ concentration^1^; Synthetic Greenland δ^18^O record^6^; Speleothem δ^18^O records of Central European^7^; Speleothem δ^13^C records of North Atlantic^8^; Chinese speleothem composite records^2^. Orange vertical bars indicate the deglacial Atlantic meridional overturning circulation (AMOC) disruptions.


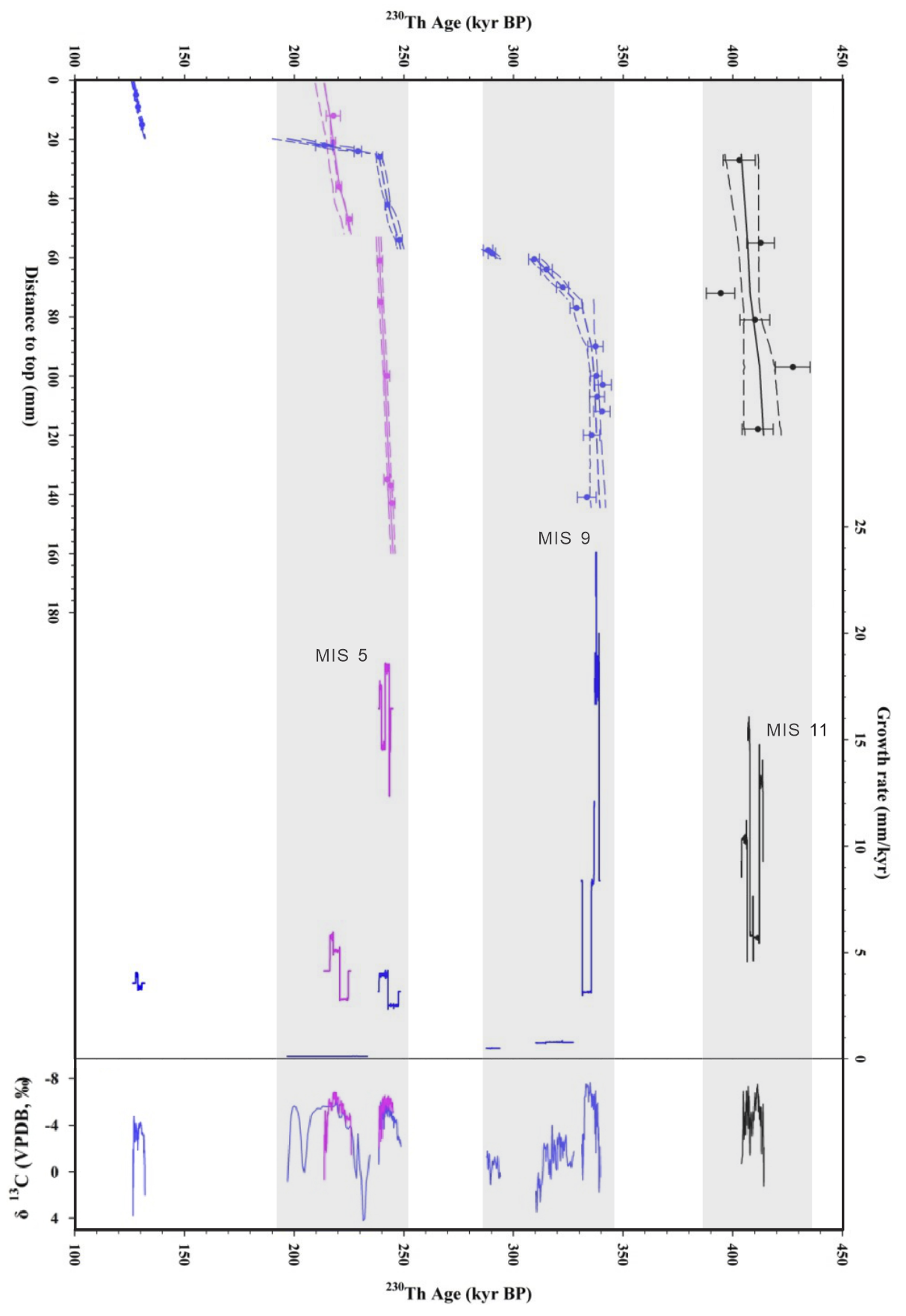


**Supplementary Fig. 6 |** **Plots of age versus depth for stalagmites 19TM-2 (black), 19TM-3 (blue)and 19TM-5 (pink) from Tianmen cave and grow rate time series.** The Tianmen speleothem δ^13^C record was shown in the bottom panel. Replotted from ref. (^4^).


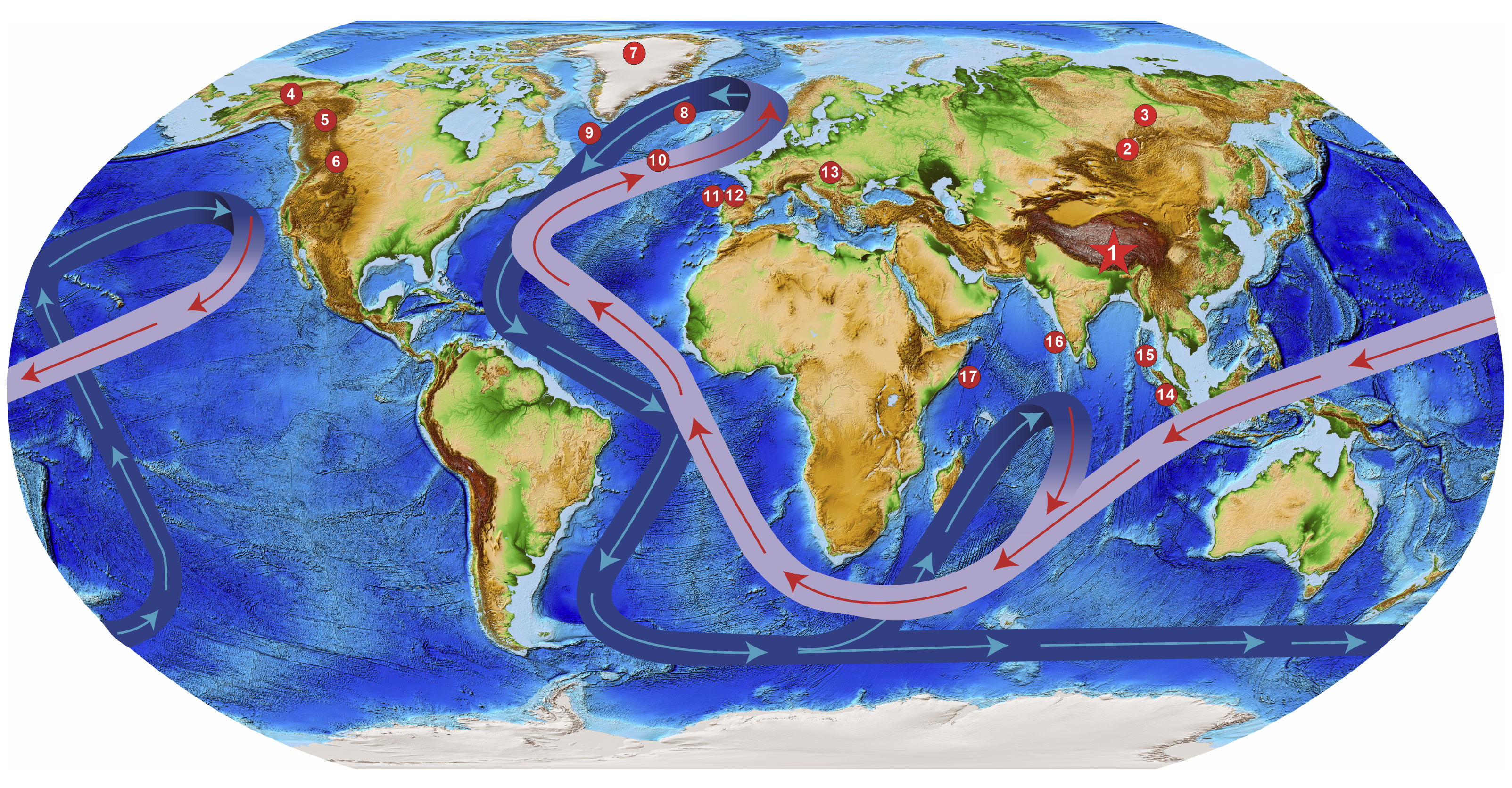


**Supplementary Fig. 7 | Tibetan Plateau cave study area compared to other sites discussed in text and sites of importance.**  1: Tianmen Cave site; 2-3: Okhotnichya, Botovskaya and Lenskaya Ledyanaya caves (Siberian permafrost region)^9^; 4-6:Caves of Northern Yukong, Nahanni and Southern Canadian Rockies (Canadian permafrost region); 7: North Greenland Ice Core Project (NGRIP)^10^; 8: ODP Site 983 (ref.^11^); 9: MD03-2665&-2664 and Site U1305; 10: Site U1304; 11: SU81-18 and MD01-2444 of Iberian Margin; 12: Northwest Iberia cave^8^; 13: Central European cave^7^. 14: SO189-39KL (off Sumatra)^12^; 15: ADM-159 (Andaman Sea)^13^; 16: SK237-GC04 (Arabian Sea)^14^; 17: MD85674 (off East Africa)^15^. Global bathymetry data are available from the GEBCO website at https://doi.org/10.5285/1c44ce99-0a0d-5f4f-e063-7086abc0ea0f (GEBCO Compilation Group, 2024).


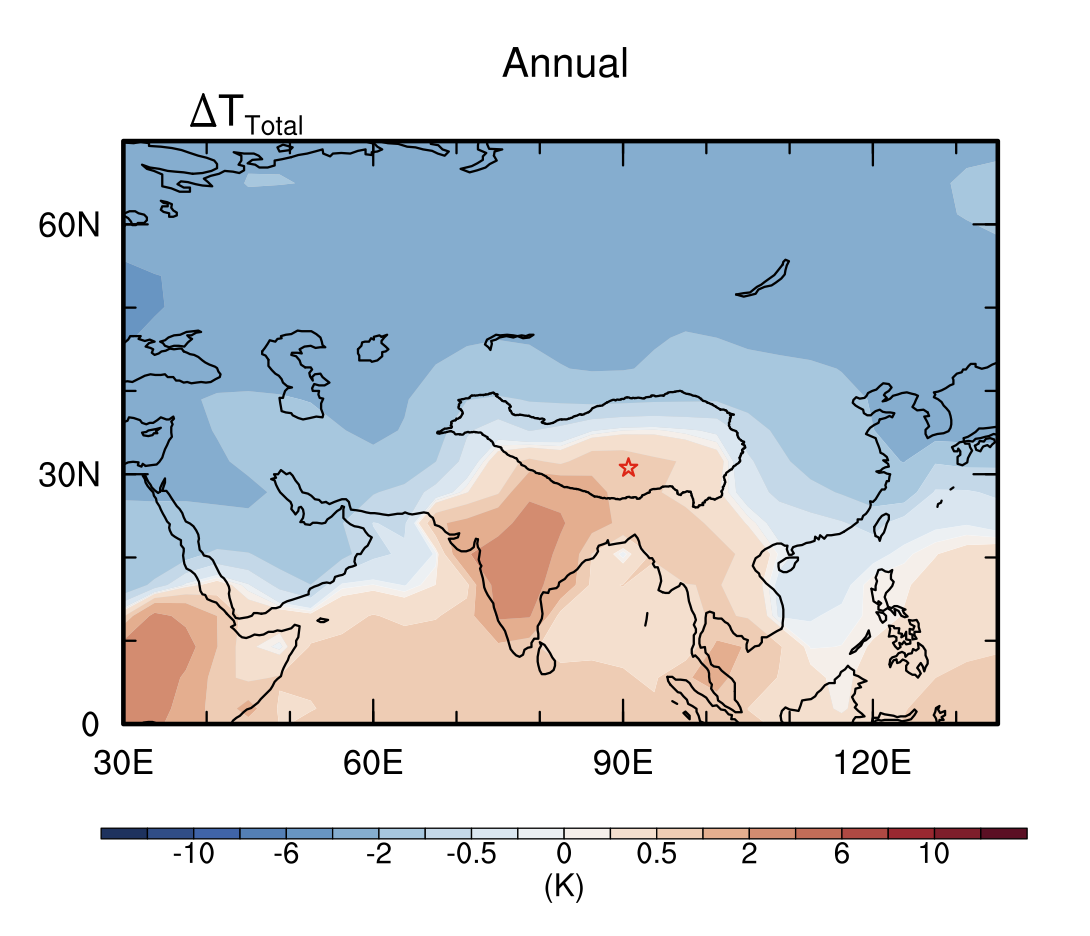


**Supplementary Fig. 8 | Tibetan Plateau annual mean temperature anomaly in response to AMOC collapse.**


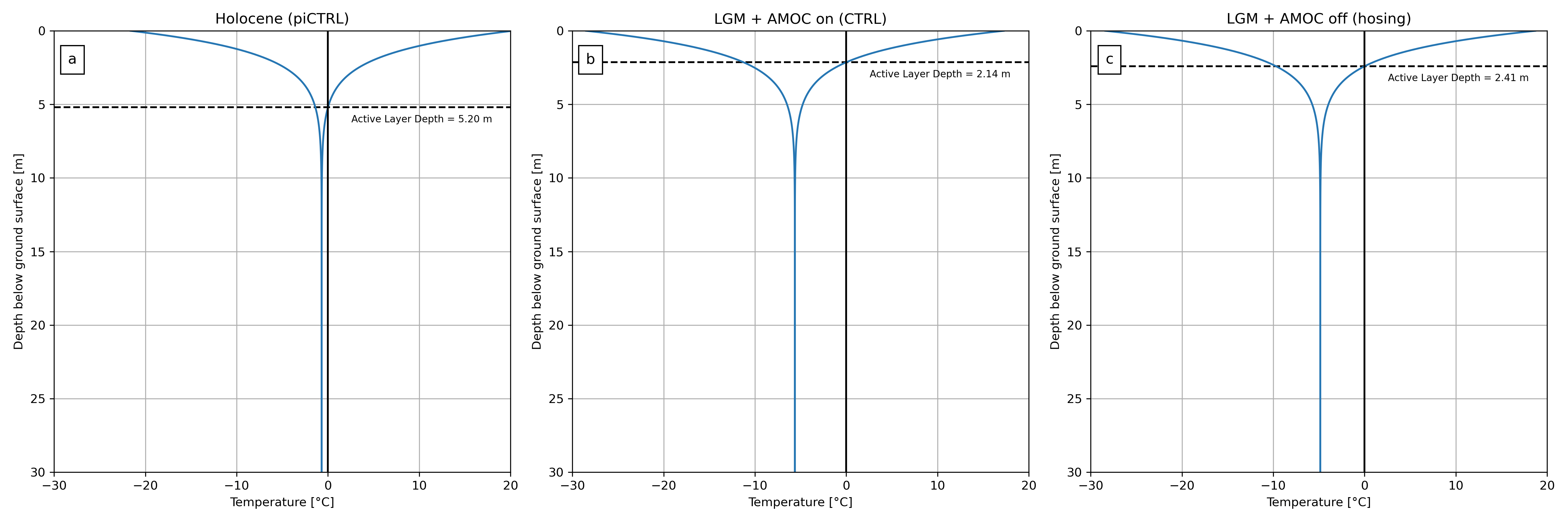


**Supplementary Fig. 9 | Permafrost temperature plots for three global climate scenarios.** (a) the Holocene, (b) the Last Glacial Maximum (LGM) with a glacial AMOC, and (c) during a “Heinrich Event” in the glacial setting where AMOC is collapsed.


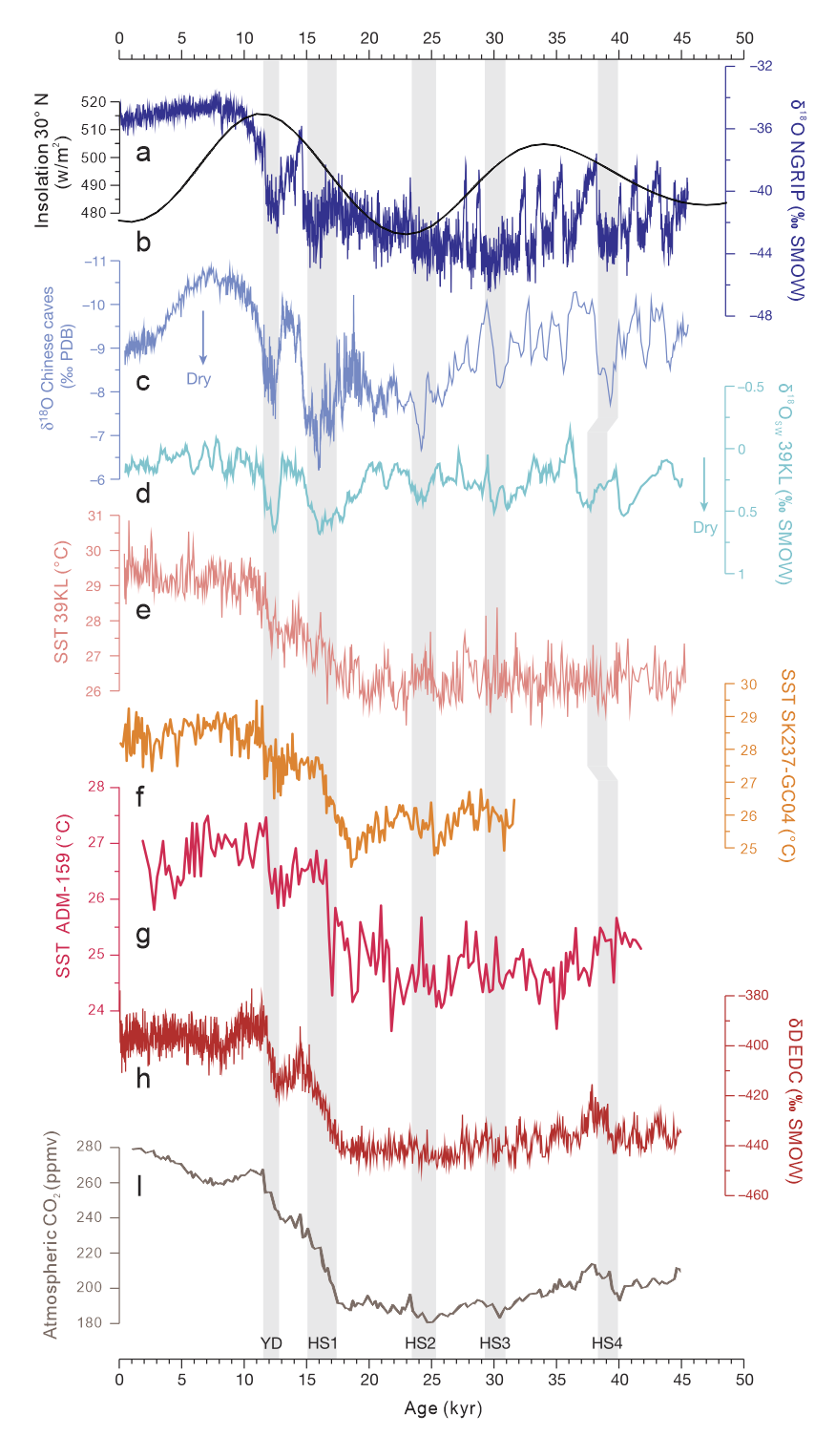


**Supplementary Fig. 10 | Comparison of South Asian hydroclimate and Indian Ocean sea surface temperature (SST) data with other records of paleoclimate.** a: δ^18^O data of Greenland ice core NGRIP^16^; b: The 30°N summer insolation^17^; c: Asian summer monsoon strength as given by a composite Chinese cave speleothem δ^18^O record^2^; d: sea-level-corrected δ^18^O_SW_ of core SO189-39KL off western Sumatra^12^; e: Sea surface temperature reconstruction at site SO189-39KL off western Sumatra^12^, f: SST reconstruction at site SK237-GC04 of Arabian Sea^14^, g: SST reconstruction at site ADM-159 of Andaman Sea^13^. h: δD data from the EPICA Dome C ice core^18^. i: Atmospheric CO_2_ record from the EPICA Dome C ice core^19^. Grey bars indicate the Younger Dryas (YD), and Heinrich stadials (HSs) 1 to 4 as recorded in North Atlantic deep-sea cores^20^.


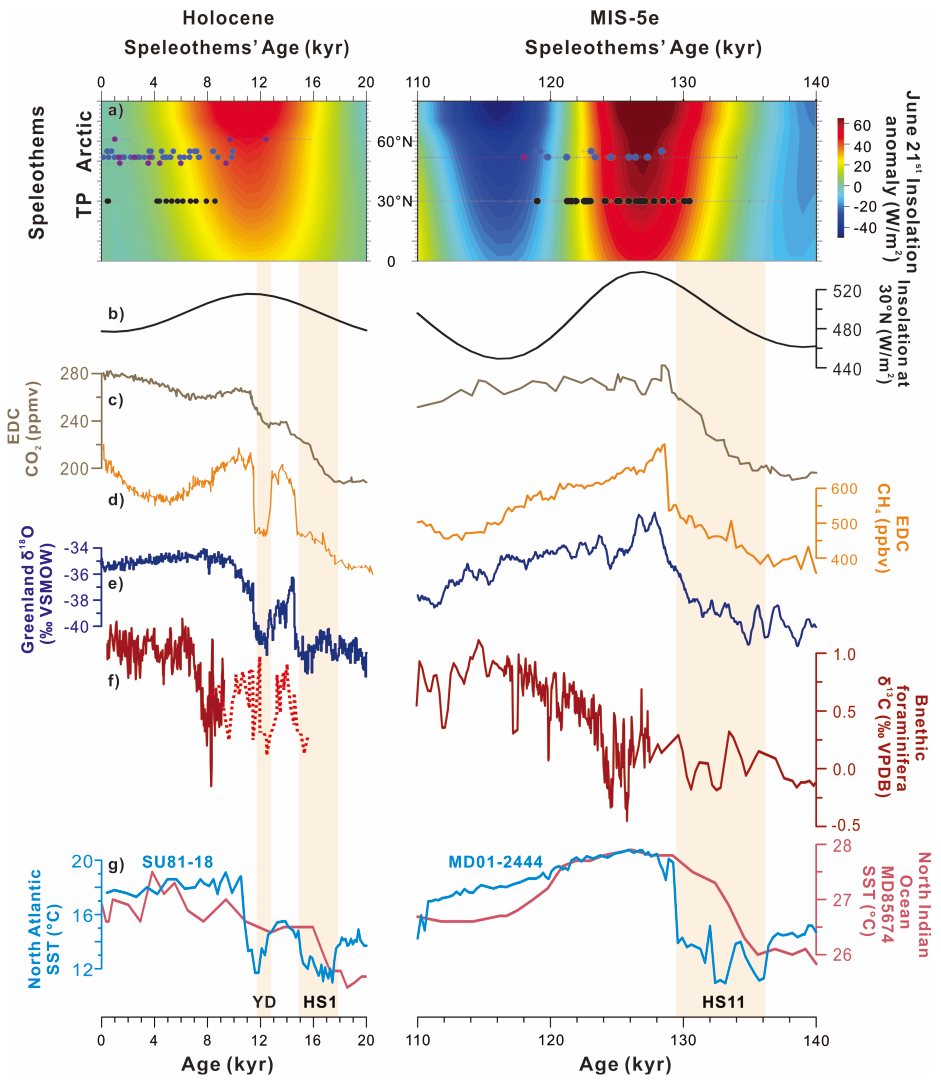


**Supplementary Fig. 11 | Forcing, climatic records and permafrost thawing across the Holocene (left) and the Last Interglacial (LIG, right).** (a) Distribution of speleothem U–Th ages (±2σ) in Canadian (purple), Siberian (blue) and Tibetan Plateau (black) caves in time (ka) and space (latitude ° N). The background is 21^st^ June insolation as anomalies relative to their average value of the last 1000 years across latitudes. (b) Summer insolation at 30 °N (orange)^17^ and EASM composite records (green)^2^. (c, d) CH_4_ and CO_2_ records, respectively, of EPICA Dome C^1,21^. (e) NGRIP Greenland δ^18^O record^10^ (Holocene). Synthetic Greenland δ^18^O record^6^ (MIS 5e). (f) *C. wuellerstorfi* δ^13^C from the Eirik Drift (MD03-2665&-2664)^22,23^ (solid line) and IODP Site U1304 in North Atlantic^24^ (dashed line). (g) Sea surface temperature reconstructions from North Indian Ocean^15^ (based on UK’37, red) and North Atlantic (Iberian margins; based on UK’37, blue)^25,26^. Orange boxes indicates deglacial Herich Events.


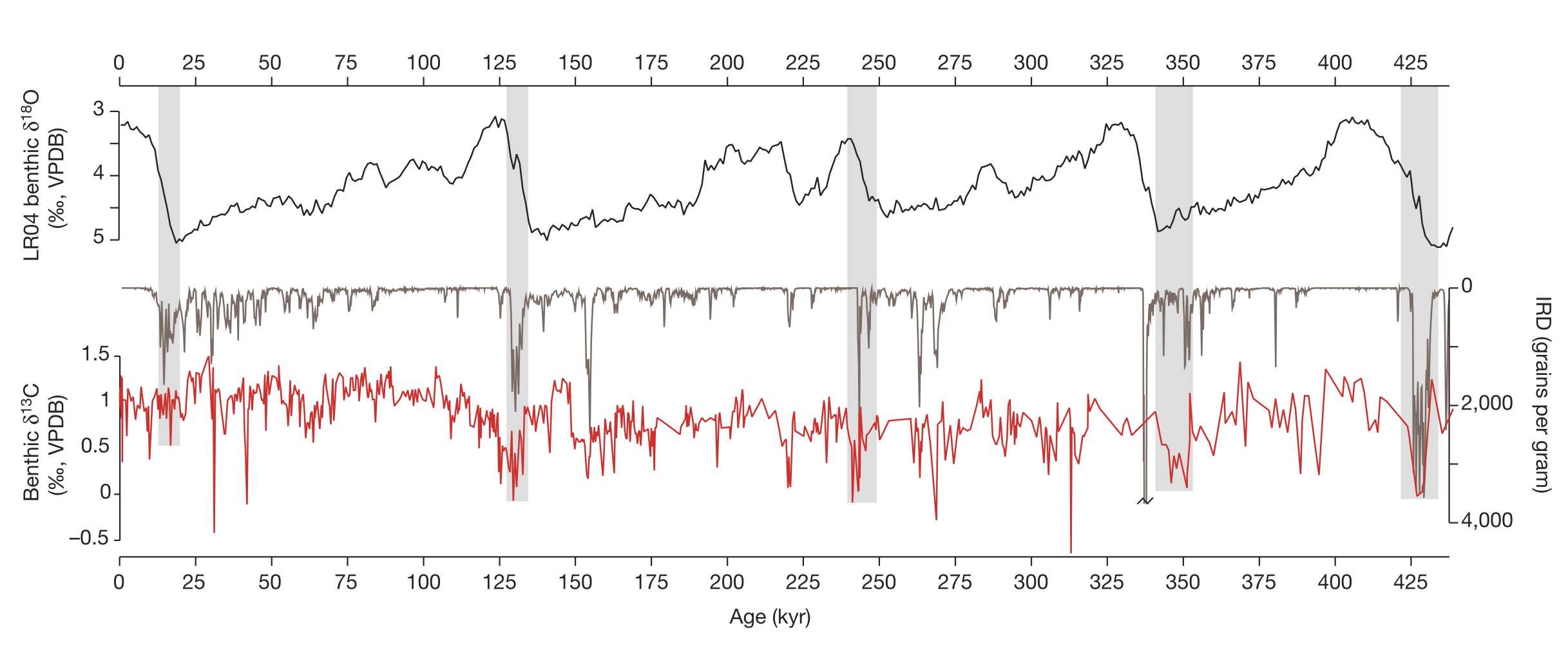


**Supplementary Fig. 12 | The** **ice rafted debris (IRD) abundance and** **carbon isotope of benthic foraminifera in the ODP Site 983**. Vertical bars indicate the terminations. Replotted from ref. (^11^).


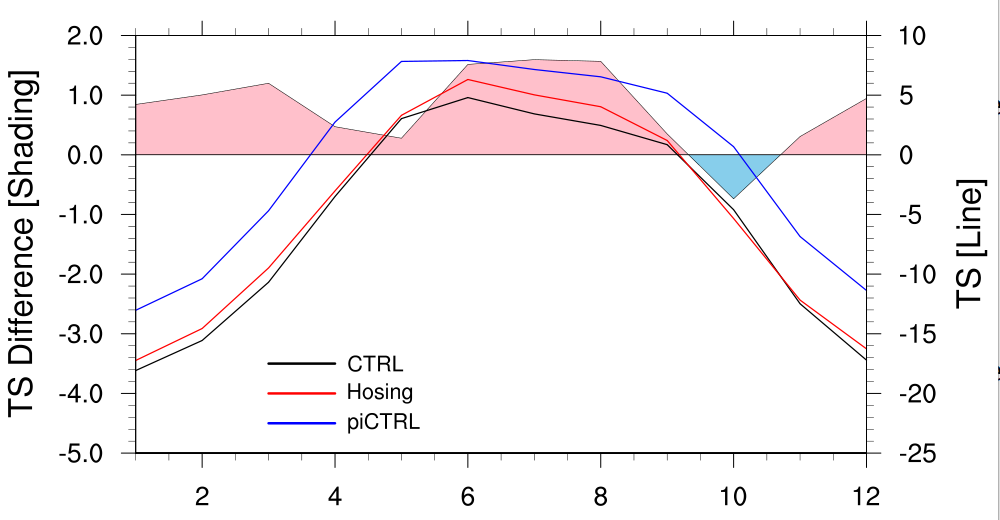


**Supplementary Fig. 13 | Simulated temperatures on Tianmen Cave in Pre-industrial (piCTRL), the Last Glacial Maximum (CTRL) and the LGM hosing (Hosing) experiments.** Summer warming anomaly in Hosing is evident in comparison to CTRL.

**Supplementary References**

1. Lüthi, D. *et al.* High-resolution carbon dioxide concentration record 650,000–800,000 years before present. *Nature* **453**, 379–382 (2008).

2. Cheng, H. *et al.* The Asian monsoon over the past 640,000 years and ice age terminations. *Nature* **534**, 640–646 (2016).

3. Berger, A. L. Long-Term Variations of Caloric Insolation Resulting from the Earth’s Orbital Elements. *Quat. Res.* **9**, 139–167 (1978).

4. Wang, H. *et al.* Orbital-scale hydroclimate variations in the southern Tibetan Plateau over the past 414,000 years. *Quat. Sci. Rev.* **291**, 107658 (2022).

5. Lisiecki, L. E. & Raymo, M. E. A Pliocene-Pleistocene stack of 57 globally distributed benthic δ18O records. *Paleoceanography* **20**, (2005).

6. Barker, S. *et al.* 800,000 Years of Abrupt Climate Variability. *Science* **334**, 347–351 (2011).

7. Koltai, G. *et al.* A penultimate glacial climate record from southern Hungary. *J. Quat. Sci.* **32**, 946–956 (2017).

8. Stoll, H. M. *et al.* Rapid northern hemisphere ice sheet melting during the penultimate deglaciation. *Nat. Commun.* **13**, 3819 (2022).

9. Cheung, M.-C., Zong, Y., Zheng, Z., Liu, Z. & Aitchison, J. C. Holocene temperature and precipitation variability on the central Tibetan Plateau revealed by multiple palaeo-climatic proxy records from an alpine wetland sequence. *The Holocene* **27**, 1669–1681 (2017).

10. Wolff, E. W., Chappellaz, J., Blunier, T., Rasmussen, S. O. & Svensson, A. Millennial-scale variability during the last glacial: The ice core record. *Quat. Sci. Rev.* **29**, 2828–2838 (2010).

11. Barker, S. *et al.* Icebergs not the trigger for North Atlantic cold events. *Nature* **520**, 333–336 (2015).

12. Mohtadi, M. *et al.* North Atlantic forcing of tropical Indian Ocean climate. *Nature* **509**, 76–80 (2014).

13. Liu, S. *et al.* Paleoclimatic responses in the tropical Indian Ocean to regional monsoon and global climate change over the last 42 kyr. *Mar. Geol.* **438**, 106542 (2021).

14. Saraswat, R., Lea, D. W., Nigam, R., Mackensen, A. & Naik, D. K. Deglaciation in the tropical Indian Ocean driven by interplay between the regional monsoon and global teleconnections. *Earth Planet. Sci. Lett.* **375**, 166–175 (2013).

15. Bard, E., Rostek, F. & Sonzogni, C. Interhemispheric synchrony of the last deglaciation inferred from alkenone palaeothermometry. *Nature* **385**, 707–710 (1997).

16. Svensson, A. *et al.* A 60 000 year Greenland stratigraphic ice core chronology. *Clim. Past* **4**, 47–57 (2008).

17. Laskar, J. *et al.* A long-term numerical solution for the insolation quantities of the Earth. *Astron. Astrophys.* **428**, 261–285 (2004).

18. Parrenin, F. *et al.* Synchronous Change of Atmospheric CO2 and Antarctic Temperature During the Last Deglacial Warming. *Science* **339**, 1060–1063 (2013).

19. Bereiter, B. *et al.* Revision of the EPICA Dome C CO2 record from 800 to 600 kyr before present. *Geophys. Res. Lett.* **42**, 542–549 (2015).

20. Hemming, S. R. Heinrich events: Massive late Pleistocene detritus layers of the North Atlantic and their global climate imprint. *Rev. Geophys.* **42**, (2004).

21. Loulergue, L. *et al.* Orbital and millennial-scale features of atmospheric CH4 over the past 800,000 years. *Nature* **453**, 383–386 (2008).

22. Kleiven, H. (Kikki) F. *et al.* Reduced North Atlantic Deep Water Coeval with the Glacial Lake Agassiz Freshwater Outburst. *Science* **319**, 60–64 (2008).

23. Galaasen, E. *et al.* Rapid Reductions in North Atlantic Deep Water During the Peak of the Last Interglacial Period. *Science* **343**, (2014).

24. Galaasen, E. V. *et al.* Interglacial instability of North Atlantic Deep Water ventilation. *Science* **367**, 1485–1489 (2020).

25. Tzedakis, P. C. *et al.* Enhanced climate instability in the North Atlantic and southern Europe during the Last Interglacial. *Nat. Commun.* **9**, 4235 (2018).

26. Bard, E., Rostek, F., Turon, J.-L. & Gendreau, S. Hydrological Impact of Heinrich Events in the Subtropical Northeast Atlantic. *Science* **289**, 1321–1324 (2000).

27. Prokopenko, A. A. *et al.* Biogenic Silica Record of the Lake Baikal Response to Climatic Forcing during the Brunhes. *Quat. Res.* **55**, 123–132 (2001).
